# Spontaneous Regression of Multiple Osteochondromas in a Patient With Hereditary Multiple Exostoses: A Case Report

**DOI:** 10.7759/cureus.88111

**Published:** 2025-07-16

**Authors:** Kazuhiro Ikeda, Shotaro Teruya, Hiromitsu Tsuge, Shinzo Onishi

**Affiliations:** 1 Department of Orthopedic Surgery, Institute of Medicine, University of Tsukuba, Tsukuba, JPN; 2 Department of Orthopedic Surgery, Kikkoman General Hospital, Noda, JPN

**Keywords:** hereditary multiple exostoses, osteochondroma, pedunculated lesion, sessile lesion, spontaneous regression

## Abstract

Hereditary multiple exostoses (HME) is an autosomal dominant disorder characterized by the development of multiple osteochondromas, primarily near the metaphyses of long bones. We report a case of HME in which multiple symptomatic lesions showed spontaneous regression. The patient was an 11-year-old boy with osteochondromas involving both distal femurs and the left forearm. At age 13, he developed discomfort and pain during squatting and forearm rotation, and radiographs revealed progressive enlargement of the lesions. Given that the physes remained open, we chose a conservative approach. At age 15, the symptomatic lesions had regressed significantly, and his symptoms resolved without surgical intervention. This case demonstrates that even symptomatic lesions in HME may regress spontaneously during growth, supporting the value of conservative management before physeal closure. Surgical decisions should be guided by the clinical course of the lesion rather than its morphology or transient symptoms.

## Introduction

Hereditary multiple exostoses (HME) is a genetic disorder that causes multiple benign osteochondromas. These lesions typically arise near the metaphyses of long bones and are inherited in an autosomal dominant pattern [1]. At birth, they are often not apparent; however, visible masses are reported in approximately 50% of patients by age 5 and in 80% by age 10 [2]. In most cases, the lesions develop near the physes and enlarge during skeletal growth, typically ceasing with physeal closure [3]. In contrast, marked enlargement after physeal closure may raise concern for malignant transformation to secondary chondrosarcoma. Malignant transformation occurs in up to 10% of HME cases [1,2]; therefore, long-term follow-up is considered essential.

Some osteochondromas may result in various complications as they enlarge, such as pain, functional impairment, skeletal deformity, neurovascular compression, and cosmetic concerns [1,2]. In such cases, surgical resection is sometimes performed. In contrast, most osteochondromas are asymptomatic, and some may even regress spontaneously [4-14]. Therefore, the standard management of osteochondromas is conservative observation. Taken together, treatment decisions are generally based on symptoms and the lesion’s longitudinal behavior, including progression or regression over time. However, most reports of spontaneous regression involve solitary osteochondromas, and such outcomes have rarely been documented in patients with HME [7,12]. As a result, the evidence base for surgical indications in HME remains limited.

To illustrate this issue, we report a case of HME in which multiple symptomatic osteochondromas regressed spontaneously without surgical intervention.

## Case presentation

**Figure 1:**
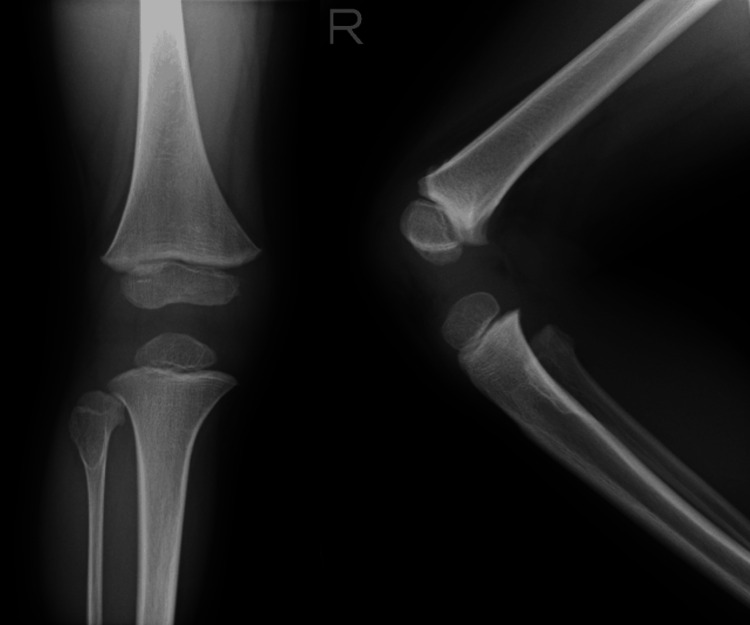
Right knee at age 3 A solitary osteochondroma was observed on the anterior aspect of the proximal fibula.

An 11-year-old boy presented to our hospital with gradually enlarging bilateral knee masses. He had been diagnosed with HME at age 3, based on a right proximal fibular osteochondroma and a maternal family history (Figure 1). However, he had not been followed regularly since the diagnosis. At age 10, he noticed enlargement of masses in the right fibula and consulted a local clinic. He was subsequently referred to our hospital the following year.

The left knee

**Figure 2:**
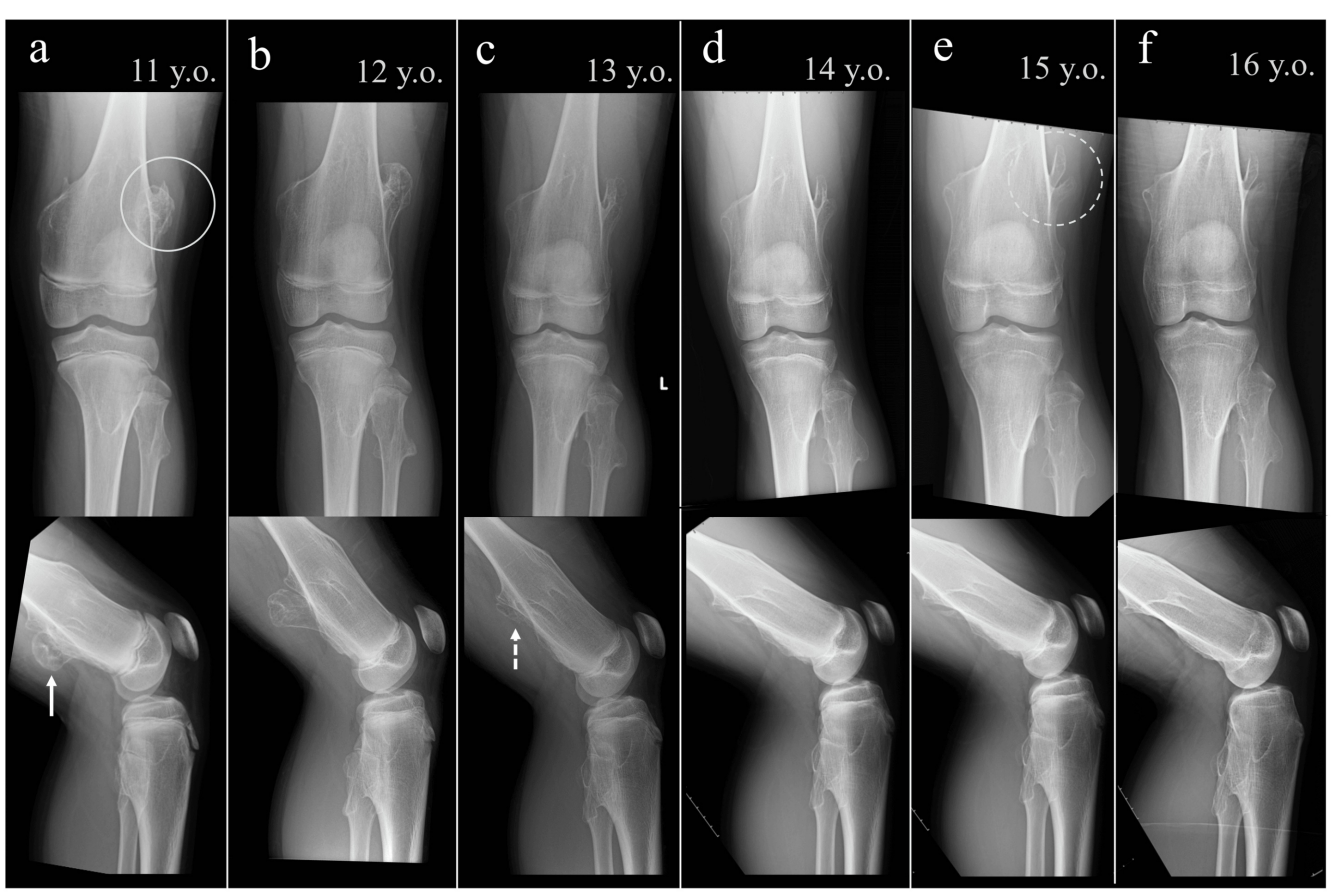
Serial radiographs of the left knee from ages 11 to 16 (a) Age 11: Sessile osteochondromas are visible on both the lateral (white circle) and posterior (white arrow) aspects of the distal femur. (b) Age 12: Both lesions increased in size. (c) Age 13: The posterior lesion regressed (dotted arrow). (d) Age 14: The lateral lesion showed a slight decrease in size (mild regression). (e) Age 15: The lateral osteochondroma transitioned from a sessile to a pedunculated morphology (dotted circle). (f) Age 16: Complete physeal closure was confirmed.

At age 11, the patient reported discomfort in the left knee, particularly during deep squatting. Physical examination revealed palpable masses over the distal femur. Radiographs demonstrated sessile osteochondromas on both the lateral and posterior aspects of the distal femur (Figure 2). At age 12, the patient began to experience pain during deep squatting. Radiographs showed enlargement of the posterior lesion, while the physis remained open. Although he expressed a desire for surgical treatment, we chose conservative management due to concern about recurrence. At age 13, his pain and discomfort with squatting had resolved spontaneously. Radiographs showed near-complete regression of the posterior lesion. Meanwhile, the lateral lesion gradually evolved from a sessile to a pedunculated, bilobed morphology but remained asymptomatic throughout the course. At age 16, the patient was asymptomatic with regard to the left knee. Radiographs confirmed complete physeal closure.

The right knee

**Figure 3:**
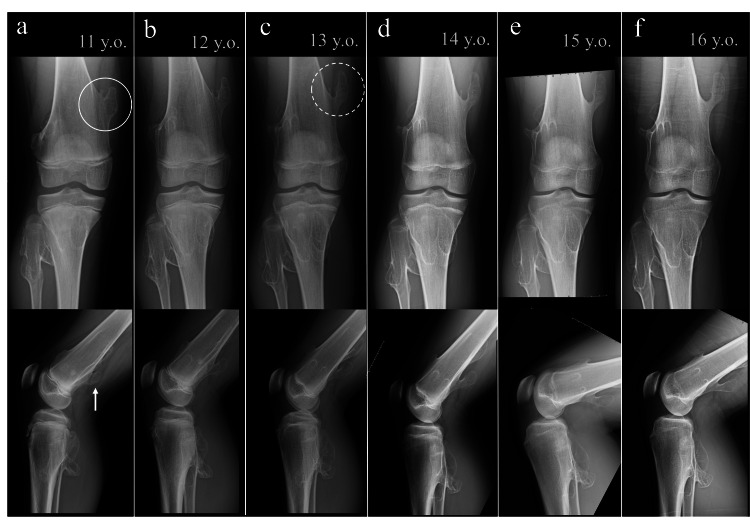
Serial radiographs of the right knee from ages 11 to 16 (a) Age 11: A sessile osteochondroma is visible on the medial aspect of the distal femur (circle), and a pedunculated osteochondroma is seen posteriorly (arrow). (b) Age 12: Both lesions gradually increased in size. (c) Age 13: The medial lesion transitioned to a pedunculated morphology (dotted circle). (d) Age 14: Both lesions remained stable. (e) Age 15: Both lesions remained stable. (f) Age 16: Both lesions persisted, and complete physeal closure was confirmed.

At age 11, subcutaneous masses were palpable over the medial and posterior aspects of the distal right femur, though the patient remained asymptomatic. Radiographs revealed a sessile osteochondroma on the medial side and a pedunculated lesion on the posterior aspect (Figure 3). At age 13, both lesions had gradually increased in size. The medial lesion had also transitioned to a pedunculated morphology.

After age 14, no further enlargement was observed. At age 16, the patient reported only a sense of mass without pain or limitation in activities of daily living.

The left wrist

**Figure 4:**
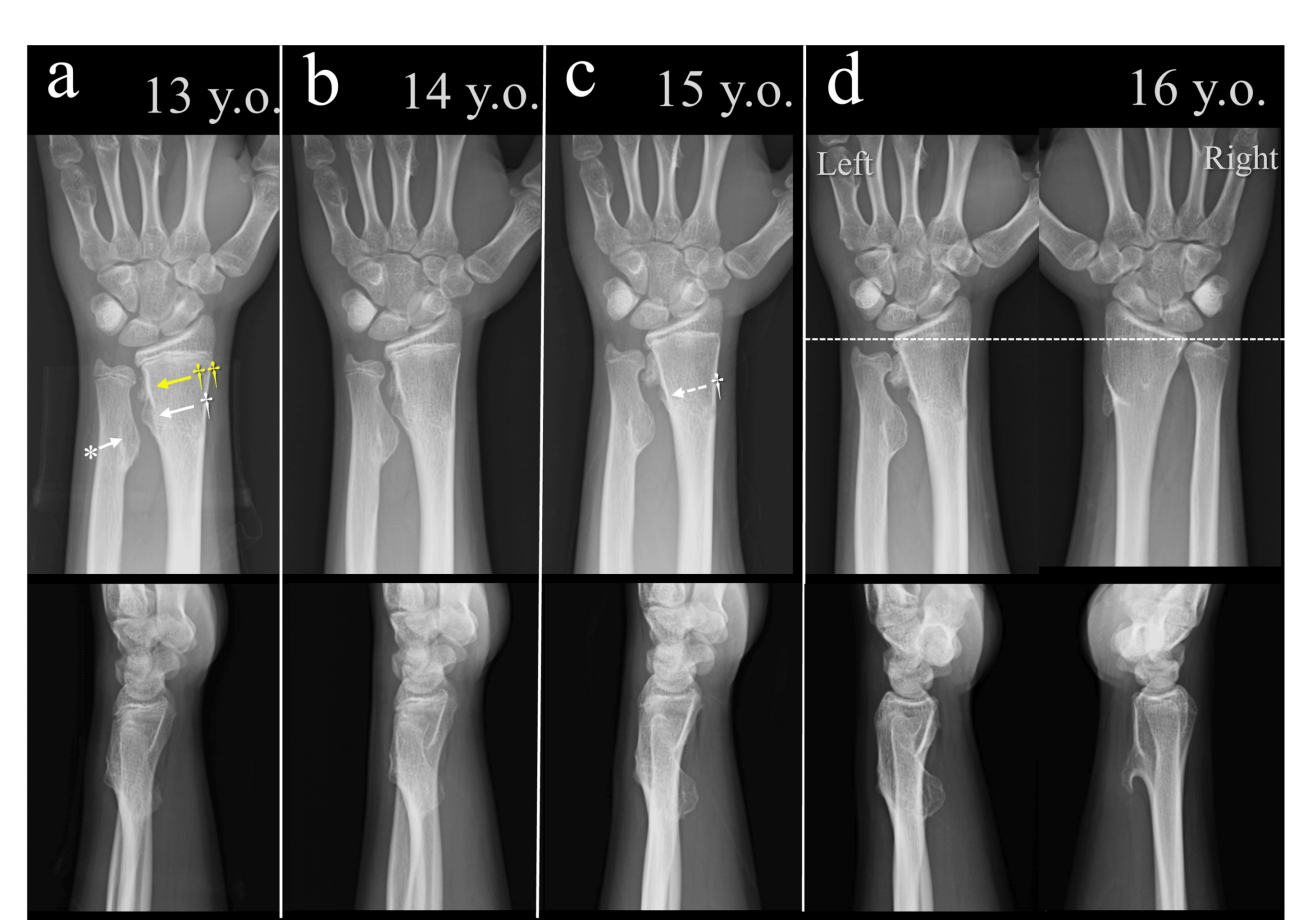
Serial radiographs of the left wrist from ages 13 to 16 (a) Age 13: A prominent ulnar osteochondroma is visible (asterisk), along with two sessile radial osteochondromas — proximal (dagger) and distal (double dagger, yellow). (b) Age 14: All lesions enlarged and became closely approximated. (c) Age 15: The proximal radial lesion regressed (dagger with dotted arrow), while the distal radial lesion continued to enlarge. (d) Age 16: The distal radial osteochondroma followed the contour of the ulnar head. Ulnar variance is shown with a white dotted line; the right wrist is included for comparison.

At age 13, the patient became aware of a mass in the distal ulna of the left wrist (Figure 4). He had no pain or limitation in forearm rotation. Radiographs showed ulnar minus variance and revealed sessile osteochondromas on the distal ulna (ulnar aspect) and distal radius (radial aspect). The radial lesions were bilobed in morphology: the proximal lobe faced the ulnar lesion, and the distal lobe was adjacent to the ulnar head. At age 14, the patient experienced increasing discomfort during forearm rotation. Both the ulnar and radial osteochondromas had enlarged and had become closely approximated. At age 15, the discomfort had resolved spontaneously. The radial lesion adjacent to the ulnar osteochondroma had regressed, whereas the distal radial lesion had continued to enlarge. At age 16, the patient remained asymptomatic with a full range of forearm rotation. The distal radial osteochondroma had grown along the contour of the ulnar head, and the congruency of the distal radioulnar joint was preserved.

## Discussion

In this case, we observed spontaneous regression of osteochondromas in HME at multiple anatomical sites over time. Notably, some of these lesions were initially symptomatic and resolved without surgical intervention. Conservative management is a reasonable primary treatment option until physeal closure, even for symptomatic osteochondromas in HME. However, regression was observed predominantly in sessile lesions, whereas pedunculated lesions showed no regression by skeletal maturity. This pattern is consistent with previous reports on solitary osteochondromas [5]. These findings suggest that conservative observation may be appropriate for symptomatic lesions with sessile morphology, whereas pedunculated lesions are less likely to regress.

The mechanism of regression appears to differ between sessile and pedunculated osteochondromas. Sessile lesions are thought to regress via the absorption theory, which proposes that the lesion is gradually resorbed through physiological remodeling after physeal closure [8]. This mechanism suggests that mechanical stress may contribute to lesion regression via adaptive remodeling [15]. The distal forearm lesion in our case demonstrated this phenomenon clearly: one lesion that might have restricted forearm rotation regressed spontaneously, while another developed distally, conforming to the contour of the ulnar head.

In contrast, pedunculated lesions appeared less responsive to mechanical remodeling. Interestingly, in our case, we observed that some pedunculated lesions developed through partial resorption at the base of sessile lesions. These findings suggest that some pedunculated lesions may not represent a distinct developmental pathway, but a morphological endpoint of remodeling in sessile lesions. Once formed, such pedunculated lesions may regress in accordance with the fracture theory, which proposes that trauma-induced disruption leads to reactive resorption via callus formation and vascular compromise [7].

This case has several limitations that warrant consideration. First, the observation period was restricted to the time surrounding physeal closure, without coverage of early childhood. As a result, we were unable to assess the incorporation theory, which proposes that the lesion is incorporated into the longitudinal growth of the parent bone before physeal closure [16]. Second, cartilage cap thickness - an important factor influencing the growth or regression of osteochondromas - was not assessed. Future observational studies should incorporate imaging modalities such as MRI or ultrasound to identify features predictive of spontaneous regression. These limitations underscore the need for both early-phase and long-term imaging-based follow-up to better understand the full natural history of these lesions.

## Conclusions

In this case, we observed spontaneous regression of sessile osteochondromas in HME at multiple anatomical sites. Notably, some of these lesions were initially symptomatic and resolved without surgical intervention. Conservative management should be actively considered as the primary treatment option until physeal closure, unless the symptoms are urgent.
